# A feasibility randomised controlled trial to evaluate the effectiveness of a novel neuromuscular electro-stimulation device in preventing the formation of oedema following total hip replacement surgery

**DOI:** 10.1016/j.heliyon.2018.e00697

**Published:** 2018-07-18

**Authors:** Thomas W. Wainwright, Louise C. Burgess, Robert G. Middleton

**Affiliations:** a Orthopaedic Research Institute, Bournemouth University, 6th Floor, Executive Business Centre, 89 Holdenhurst Road, Bournemouth, BH8 8EB, UK; b Healthdecoded Ltd, c/o Hilldean, Manor House, Broadstone, BH18 8AS, UK

**Keywords:** Biomedical engineering, Physiology, Rehabilitation, Surgery

## Abstract

**Aim:**

The aim of this feasibility study was to investigate the potential role of a novel neuromuscular electrical stimulation (NMES) device in preventing the formation of oedema following total hip replacement (THR).

**Methods:**

Successive primary THR patients were recruited into a randomised controlled trial. Participants were randomised to wear either the NMES device or compression stockings continually from post-surgery until discharge.

The main outcome measure was presence of lower limb oedema, assessed by taking measurements of the circumference of the ankle, knee and thigh on the operated leg and non-operated leg, pre-operatively, post-operatively, at two days post-operatively and every day until discharge. Secondary objectives were to compare adverse events, the presence of asymptomatic and symptomatic deep vein thrombosis (DVT) and device tolerability between groups.

**Results:**

Data from 40 participants were analysed (NMES (n = 20), compression stockings (n = 20)). The NMES group had significantly less oedema and the device was found to be tolerable and safe.

**Conclusion:**

The results of this study suggest that the NMES is a safe and well tolerated alternative to compression stockings, which should be considered by clinicians seeking the additional benefit of reducing post-operative oedema. In addition the NMES device should be considered as part of a DVT prophylaxis.

## 1 Introduction

Total hip replacement (THR) is a common and successful surgical solution for the treatment of osteoarthritis of the hip. The procedure has demonstrated positive results for patients and the development of surgical techniques, pain management strategies and enhanced recovery after surgery (ERAS) pathways have improved outcomes further (Ibrahim et al., 2013). Length of stay has reduced (Den Hartog et al., 2013) and return to function has been accelerated with no increase to post-operative complications or re-admission rates (Husted et al., 2008). In addition, outpatient THR has been considered feasible in selected (Den Hartog et al., 2015) and unselected patients (Gromov et al., 2017). However, some issues still remain for patients post-surgery. Husted et al. (2011) found the main clinical reasons for delayed discharge following THR to be pain, dizziness and general weakness. Decreased muscle function or weakness immediately post-op is unlikely to be due to atrophy alone, and the formation of oedema post-operatively is widely accepted to lead to pain and to exacerbate loss of muscle function. Clinically, oedema is described as an abnormal build-up of interstitial fluid in the body that is enough to produce palpable swelling (Kerchner et al., 2008). In addition to surgical trauma, decreased mobility immediately post-operatively can lead to a reduction in venous return, preventing the movement of fluid from tissues back into blood vessels (Kerchner et al., 2008). This creates an imbalance between capillary filtration and lymph drainage, leading to swelling.

Currently, there is limited evidence that describes the effect of oedema on recovery, or what percentage of patients develop lower-limb oedema following THR. Traditional treatment methods for oedema following total knee replacement (TKR) may include cooling or cryotherapy, early ambulation, elevation of limbs, compression stockings and massage to help stimulate the release of excessive fluids.

An alternative treatment method is neuromuscular electrical stimulation (NMES), which stimulates nerves to activate muscle, and has been shown to enhance venous blood flow and reduce oedema in non-hip replacement populations (Doran and White, 1967; Dejode et al., 1973; Faghri et al., 1997; Broderick et al., 2010; Tucker et al., 2010). However, the clinical application of NMES for post-surgical oedema prevention has been limited until now due to concerns over perceived discomfort and the impractical design of devices (Broderick et al., 2011). A number of studies have demonstrated that NMES significantly improves blood flow in the deep veins (Faghri et al., 1997; Broderick et al., 2010; Tucker et al., 2010; Browse and Negus, 1970; Dejode et al., 1973), and the NMES device has been found to increase blood flow in the deep veins of the calf (Griffin et al., 2016). Recent developments in the design of NMES devices have significantly increased patient comfort and tolerance of NMES by allowing effective stimulation with lower current density and pulse duration (Broderick et al., 2011). These improvements in comfort have been further developed by utilising indolent nerve stimulation in place of direct muscle stimulation (Browse and Negus, 1970).

The primary objective of this study was to evaluate whether an NMES device is more efficient than compression stockings (Thromboembolic deterrent stockings (TEDS)) in preventing the formation of oedema following THR surgery. Compression stockings were chosen as the comparator as they are routinely used in the study site for the prevention of lower limb oedema and DVT. Secondary objectives were to compare the formation of asymptomatic and symptomatic DVT, to compare device acceptability, tolerability and compliance, and to compare device safety by monitoring for adverse events.

## 2 Methods

This was a randomised open label study comparing NMES and compression stockings with full ethical approval granted by the National Research Ethics Service (NRES) (REC reference 13/LO/0059, Protocol number FKD-TEDS-001, IRAS project ID 117650). The study was conducted in accordance with the principles of the Declaration of Helsinki (Ethical Principles for Medical Research Involving Human Subjects) and in compliance with European Standard ISO 14155:2011 Clinical Investigations of Medical Devices for Human Subjects – Good Clinical Practice. It was registered on ClinicalTrials.gov (Identifier: NCT01935414). The study is reported according to CONSORT guidelines for reporting randomised controlled trials (CONSORT, 2017).

Consecutive primary THR operations performed by a single surgeon at a private hospital were screened for eligibility in accordance with the inclusion and exclusion criteria detailed in Table 1. Recruitment continued until 40 patients had completed the trial. Eligible patients who consented to take part in the study were randomised by a clinical researcher with a 1:1 allocation via a sealed envelope prepared by an independent Clinical Research Organisation. All participants followed the standard care pathway for hip replacement and received the standard care for oedema prevention before surgery, during surgery and on discharge. The only difference in treatment between the two groups was that from post-surgery until discharge the participants were randomised to receive either the NMES device or compression stockings.

**Table 1:** Details of inclusion and exclusion criteria.

Inclusion criteria
• Aged 18 years of age and over • Free of significant abnormal findings as determined by medical history. • Has not used any medications (prescribed or over-the-counter including herbal remedies) judged to be significant by the Principal Investigator during the ten (10) days preceding enrolment. • Able to understand the Patient Information Sheet and willing to sign the written Informed Consent Form. • Able and willing to follow the protocol requirements.
**Exclusion criteria**
• Are requiring hip revision surgery • History or signs of previous deep or superficial vein thrombosis/pulmonary embolism. • Evidence of asymptomatic DVT by Duplex Ultrasound • Peripheral arterial disease (ABPI <0.8), varicose veins or lower limb ulceration or ischemia. • Significant varicose veins, phlebitis or lower limb ulceration or ischemia. CEAP Grade 4–6. • Recent surgery within the last 3 months (such as abdominal, gynaecological, hip or knee replacement). • Recent trauma to lower limb. • Chronic Obesity (BMI Index >40 kg/m^2^). • Pregnancy. • Significant history of following diseases • Cardiovascular: Recent MI (<6 months) • Percutaneous Coronary Intervention (PCI) with stent <3 months for Bare Metal Stent (BMS) and <12 months for Drug Eluding Stent (DES) • Moderate to severe CCF, uncontrolled AF • Neurological: Stroke, Hemiplegia/Paraplegia, Myopathies • Significant dermatological conditions affecting lower limbs resulting in broken or inflamed skin particularly at the site where the device is to be fitted. • Clinically significant haematological conditions i.e. coagulation disorders, sickle cell disease • Psychiatric disorders • On LMWH/Heparin (Prophylactic/therapeutic doses) or Warfarin or warfarin stopped recently and replaced by LMWH/Heparin • Long term steroid with dermatological changes • A pulse rate of less than 40 beats/minute • A sitting systolic blood pressure >180 and <100 mmHg and/or a sitting diastolic pressure of >100 mmHg. • Any significant illness during the four (4) weeks preceding the hip replacement surgery. • Participation in any clinical study during the eight (8) weeks preceding the screening period

The geko™ NMES device was used for the study, which is small disposable and internally powered and can be applied externally to the leg. The device is manufactured by Firstkind Ltd., High Wycombe, United Kingdom. It is self-adhesive and applied to the outer/posterior aspect of the knee. This positioning enables integral electrodes to apply a stimulus to the lateral popliteal nerve (often additionally termed the common peroneal) which branches from the sciatic nerve. These nerves control the contraction of several muscles in the lower leg. The stimulation of these nerves by the geko™ device causes the muscles to contract isometrically and will not affect normal movement of the limb nor mobility of the patient. Contraction of the lower leg muscles increases blood flow from the lower limbs back to the heart thus increasing venous return, local blood circulation and help prevent venous thrombosis (Tucker et al., 2010). Saphena^®^ anti-embolism compression stockings fitted in accordance to manufacturer instructions were used for the study, with a pressure of 18 mmHg = /−20% administered to the ankle, 14 mmHg ± 20% administered to the calf and 9 mmHg ± 20% to the thigh. Both devices were worn continually from post-surgery until discharge. In accordance with the manufacturer's instructions for use, the NMES device was changed each day.

### 2.1 Data collection

Data were collected prior to surgery, immediately following surgery, on each post-operative day until discharge, and at 6 weeks following surgery. At each time point adverse events and device deficiencies were monitored.

Oedema was examined by taking measures of the circumference of the ankle, knee and thigh on the operated leg and non-operated leg, in the supine position, pre-operatively, post-operatively (prior to fitting of either the NMES or compression stocking devices), at two days post-operatively and every day until discharge (typically day four in this study population). The position the measurement was taken was marked with an indelible marker to ensure that measurements were always recorded on the same part of the ankle/leg, and the same staff member completed all measurements.

In addition to oedema evaluation, Duplex Ultrasound was used to assess the presence or absence of asymptomatic DVT in order to ensure the use of NMES over compression stocking did not increase DVT risk. All scans were completed by a consultant radiologist who examined the common femoral vein, superficial femoral vein, popliteal vein, gastrocnemius veins, soleal veins, posterior tibial veins and peroneal veins for patency, compressibility and the presence or absence of flow.

Evaluation of the acceptance and tolerability of both compression stockings and the NMES device was completed by the administration of a Likert Scale questionnaire designed to assess the level of pain/discomfort felt by the patient on a scale of 1–5.

### 2.2 Statistical methodology

This feasibility study was deemed a necessary first step for the calculation of a suitable sample size for a future comprehensive study due to the shortage of existing suitable data for effect size. Incidence rates for oedema vary widely between patient groups and clinical settings, and the effect size for the NMES device for reducing the incidence of oedema was as yet unknown.

For oedema data, graphs were plotted of circumference versus time and the gradients compared using a t-test. The frequency of asymptomatic DVT was recorded for each group at 0 hrs (baseline scan) 48 hrs post-operatively, discharge and Week 6. Rates of DVT (asymptomatic or symptomatic) were compared at each time-point, as well as the differences between time-points. Tolerability data for each intervention was collected on discharge, and measured using a Likert 1–5 scale. Interventions were subsequently compared with Mann-Whitney U-test. Additionally, safety was assessed for each intervention by the recording of adverse events. Additional data collected such as basic demographic information were checked by student's t-test to determine any differences between groups.

## 3 Results

**Fig. 1:**
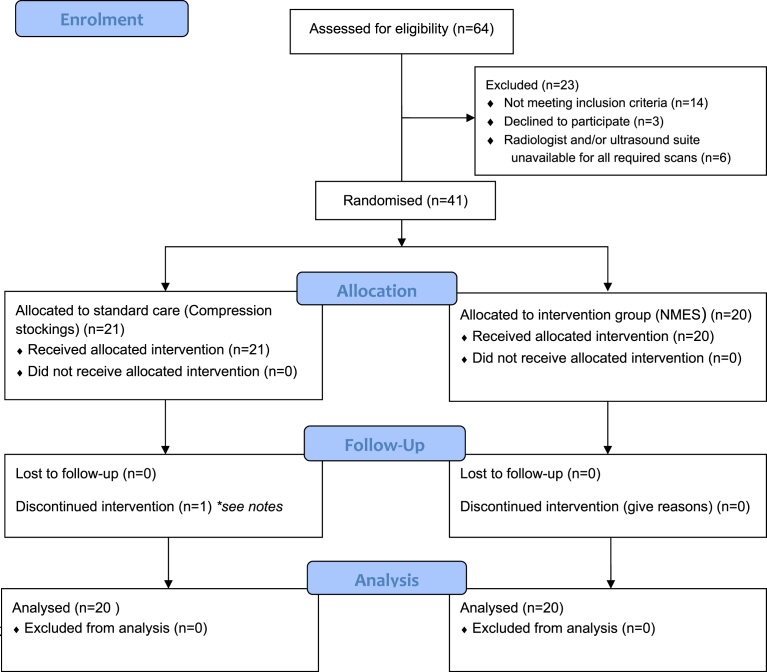
Participant flow diagram. Successive patients undergoing primary THR at the hospital were recruited between 28/08/13 and 09/07/2014 until 40 patients had completed the trial. Follow up of the last patient was completed on 11/08/14. Notes – Subject 021 experienced a “clunking sound” from the operated hip 2 days following the index surgery. An x-ray was performed but was inconclusive, so the subject underwent an exploratory operation. There were no abnormal findings during the procedure, but the operating surgeon (Chief investigator) changed the head of the prosthesis. There was no relation between the SAE and the study device. The investigator made the decision to withdraw this subject from the study prior to discharge.

Successive patients were recruited between 28/08/13 and 09/07/14 until 40 patients had completed the trial, and follow up of the last patient was completed on 11/08/14. Forty-one patients scheduled for elective total hip replacement were enrolled into the study; one subject (021) was withdrawn due to withdrawal of consent as a result of an adverse event (See Fig. 1). In general both study groups were well matched. A greater proportion of males were in the compression stockings group. The side operated on was more evenly distributed in the compression stockings population, whilst the right side received surgery in 70% of the NMES participants (See Table 2).

**Table 2:** Baseline demographic and clinical characteristics.

	NMES Mean ± 95% CI	Compression stockings Mean ± 95% CI	T-test or *Fishers exact p value
Age (years)	67.2 ± 9.2	67.8 ± 11.9	0.87
Sex	14 Female/6 Male	5 Female/15 Male	*0.01
BMI (kg/m^2^)	27.2 ± 4.3	26.5 ± 2.7	0.56
Treated leg	6 left, 14 right	8 left, 12 right	*0.74

### 3.1 Oedema

There were no significant differences seen between the pre-operative swelling measured before the applications of the NMES device or compression stockings at the ankle (P = 0.3), knee (P = 0.12) or thigh (P = 0.73) in the operated leg. Furthermore there were no significant differences seen between the post-operative swelling measured before the applications of the NMES device or compression stockings, at the ankle (P = 0.5), knee (P = 0.11) or thigh (P = 0.44) in the non-operated leg.

**Fig. 2:**
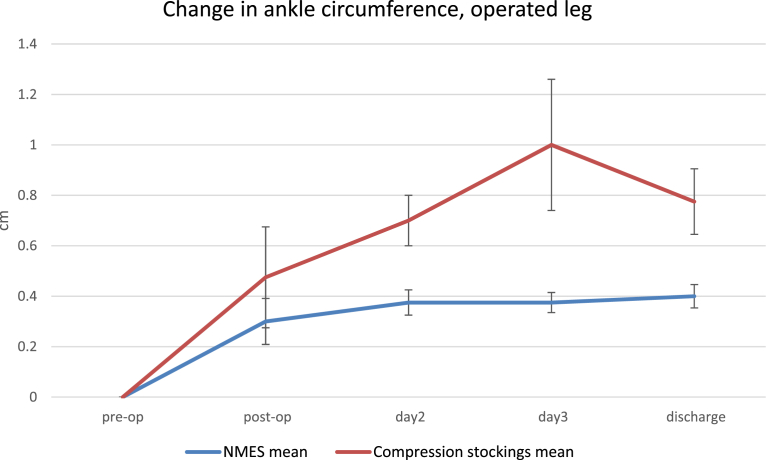
Ankle circumference in the operated leg.

Fig. 2 shows the mean ankle circumference during the post-op period to discharge relative to pre-op value for the NMES and compression stockings groups. Both groups exhibited an increase in this parameter, relating to ankle swelling during the post-op period, after application of the device. The compression stocking group ankle circumference increased by 0.48 cm ± 0.2 cm between pre-operative and post-operative measurements, the circumference peaked at day 3 (1 cm ± 0.4 cm) before dropping to 0.8 cm ± 0.3 cm on the day of discharge. The NMES group also saw an increase between the pre-operative measurements of ankle circumference and the post-operative measure prior to the device being fitted (0.3 cm ± 0.1 cm). The NMES group ankle circumference peaked on the day of discharge at 0.4 cm ± 0.1. The compression stockings group ankle circumference increased more than the NMES group though this did not reach significance (p = 0.27).

**Fig. 3:**
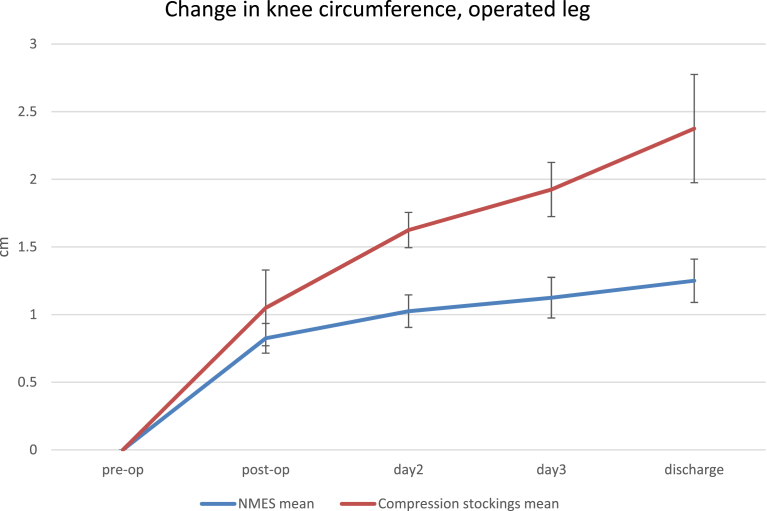
Knee circumference in the operated leg.

Similar results were seen for knee circumference (Fig. 3). Both groups show an increase between pre-operative and post-operative measures before device fitting (NMES 0.8 cm ± 0.1 cm, compression stockings 1.1 cm ± 0.3) which continued through to discharge (NMES 1.3 cm ± 0.2 cm, compression stockings 2.4 cm ± 0.5 cm), but in this case, the increase for the NMES group was significantly smaller than for the compression stockings group (p = 0.02).

**Fig. 4:**
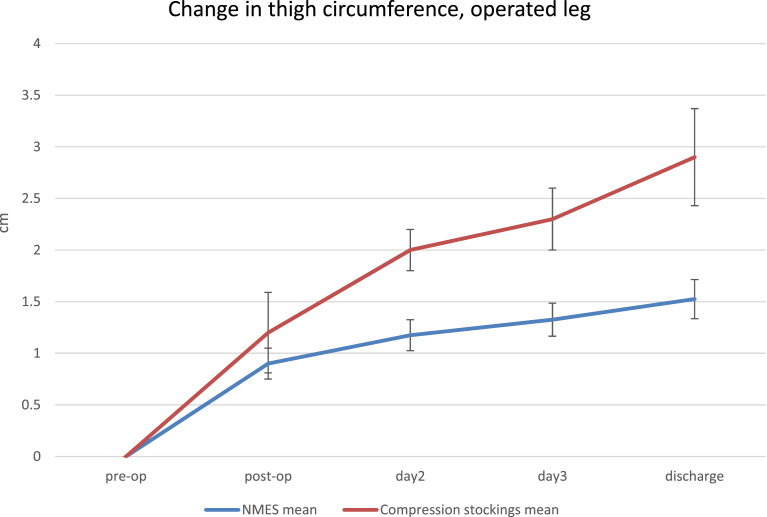
Thigh circumference in the operated leg.

Similar results were seen for the thigh measurements as shown in Fig. 4. Both NMES and compression stockings groups displayed significant increases in thigh circumference between pre-operative and post-operative measures prior to fitting the devices (0.9 cm ± 0.2 cm [p = 0.004] and 1.2 cm ± 0.4 cm [p = 0.002]). The circumference was highest in both NMES and compression stockings groups on the day of discharge (1.5 cm ± 0.3 cm and 2.9 ± 0.6 cm respectively). A significantly smaller increase in thigh diameter pre-op to discharge for the NMES group (1.5 cm ± 0.3 cm) as compared to the compression stockings group (2.9 cm ± 0.6 cm [p = 0.02]) was observed.

The treatment devices were worn on both legs by all study subjects. In the non-operated leg, no significant changes were seen in ankle circumference from immediate post-op to discharge for either group.

At the knee, the NMES group showed a non-significant decrease (p = 0.19) in non-operated knee circumference immediately post-op (0.33 cm ± 0.1 cm) to discharge (0.25 cm ± 0.1 cm), while the compression stockings group showed a non-significant increase (0.23 cm ± 0.2 cm immediately post-op to 0.43 cm ± 0.2 cm [p = 0.057]). The difference between the NMES and compression stockings groups on this parameter was significant (p = 0.016) at discharge.

Similarly, at the thigh, the NMES group showed a non-significant decrease (p = 0.095) in thigh circumference of the non-operated leg (post-operative 0.55 cm ± 0.2 cm to 0.1 cm ± 0.3 cm on the day of discharge). However, the compression stockings group showed a significant (p = 0.021) increase in thigh circumference of the non-operated leg after application post-op (0.38 cm ± 0.3 cm) to discharge (0.75 cm ± 0.3 cm). The difference between the NMES and compression stockings groups was highly significant (p = 0.006) at discharge.

### 3.2 Incidence of symptomatic or asymptomatic DVT

There were no symptomatic or asymptomatic DVTs observed via ultrasound occurring at any time point up until 6 weeks in either the NMES or compression stockings groups. There were no VTE, PE, stroke or deaths recorded during the study in either NMES or compression stockings treatment groups.

### 3.3 Patient rated outcome measures

Participants were asked at the end of their hospital stay the following question “*When compared to a blood pressure cuff inflated around your upper arm, how does the device/stocking feel?*” in order to gain an understanding as to their tolerance of the device. Over 80% of all participants i.e those randomised to NMES and compression stockings reported minimal or no sensation from their devices. There were n = 5 compression stockings participants who reported either mild or severe discomfort caused by their devices, compared with only n = 2 NMES participants reporting the same, this however did not reach significance (Fisher Exact Test, P = 0.28), see Table 3 below.

**Table 3:** Patient reported comfort.

How does the device feel	Group		
	NMES	Compression stockings	Total
1 = No sensation	1 (2.5%)	4 (10%)	5 (12.5%)
2 = Minimal sensations	16 (40%)	12 (30%)	28 (70%)
3 = Mild discomfort	2 (5%)	4 (10%)	6 (15%)
4 = Moderate discomfort	0 (0%)	0 (0%)	0 (0%)
5 = Severe discomfort	0 (0%)	1 (2.5%)	1 (2.5%)

### 3.4 Adverse events

A total of 19 adverse events (AEs) were reported during the study. The majority of these were post-operative pain (n = 12, 63.2%). None had causality related to the device. From these 19 adverse events, 4 were classified as serious adverse events (SAEs). None of the SAEs were considered Unanticipated Serious Adverse Device Effects (USADEs). No device deficiencies were reported throughout the duration of the study.

## 4 Discussion

This feasibility study was set up to provide data to adequately power a larger, multi-centre study. As such, the statistical powering and sample size of 40 participants was not designed to identify differences in oedema or DVT incidence between the interventions. Whilst the sample size is small, the fact that this was a single surgeon series, within the same hospital means that a high degree of pathway and treatment standardisation was possible. This is an important factor when assessing outcomes such as oedema or DVT which may have multi-factorial contributing factors.

The NMES device provided a potentially highly significant clinical benefit of helping to limit the amount of post-operative oedema. The NMES device performed significantly better than compression stockings in treating post-operative oedema of the knee and thigh that occurs following THR in both the operated and non-operated legs. It is acknowledged that the measurement technique for oedema evaluation has limitations, but given the circumference changes reported, and the steps to ensure reliability (same assessor and marked points of measurement), it is highly likely the differences reported are real. Any future studies, most seek to confirm these oedema findings, and aim to establish the clinical significance of reduced oedema, by linking to functional outcomes such as muscle function and functional ability.

In addition, the data provides extremely encouraging data and support for the use of the geko^TM^ device which is a new and novel form of NMES. This is because there were no AEs or SAEs attributable to the NMES device and no device deficiencies were reported. In addition, all participants were able to tolerate wearing the NMES devices continuously for the duration of their post-operative recovery in hospital 24 hours per day. These results suggest that the NMES device is both safe and robust enough to use in the clinical setting.

The positive patient tolerability data for the NMES device is especially interesting given that patient compliance with compression stockings has been previously reported to be low owing to issues of discomfort, difficulty applying and appearance. In addition, the use of compression stockings is contra-indicated in patients with leg oedema, cardiac failure, peripheral vascular insufficiency, and venous ulceration, which can limit their applicability, particularly in elderly populations who frequently have hip replacements procedures. Indeed, for patients where mechanical/pharmacological methods of prophylaxis are impractical or contraindicated, NICE medical technologies guidance recommend the adoption of NMES (Summers et al., 2015) and this study further supports the NICE position. Incorrectly fitted compression stockings have also been associated with ischemia and increased risk of DVT and a recent study has found a significant increase in skin problems, including breaks, ulcers, blisters, and necrosis in patients who were fitted with compression stockings. Future work will need to consider the cost of compression stockings compared to the NMES device given the current economic challenges within the National Health Service (NHS). It is acknowledged that compression stockings may be cheaper to purchase, however health economists should consider the overall patient cost in regards to readmissions, complications and additional rehabilitation needs.

Finally, length of stay (LOS) was not used as an outcome measure within this study as all participants stayed in hospital for four days due to funding arrangements for patient care used within the hospital. However, using NMES to reduce the formation of post-operative oedema may have a positive effect on LOS. The relationship between reduced oedema and improvement to muscle strength has not yet been proven. However we theorise that reducing oedema will encourage mobilisation and thus improve muscular strength and achievement of discharge criteria and early functional recovery.

## 5 Conclusion

Recent studies on fast-track (or Enhanced Recovery after Surgery) cohorts suggest that there may be an increased role for non-pharmalogical devices that can be used whilst walking (Jorgensen and Kehlet, 2017). This is the first randomised controlled trial to evaluate the effectiveness of the geko™ device. The results of this study suggest that the NMES device is safe, well tolerated by patients and may be more effective than compression stockings in reducing the formation of post-operative oedema. In addition and in respect to DVT prophylaxis, our findings suggest that the NMES device could be used as adjunctive to chemoprophylaxis or, as recommended by NICE, as a standalone intervention when other methods are impractical or contraindicated.

## Declarations

### Author contribution statement

Thomas Wainwright: Conceived and designed the experiments; Performed the experiments; Analyzed and interpreted the data; Contributed reagents, materials, analysis tools or data; Wrote the paper.

Louise Burgess: Contributed reagents, materials, analysis tools or data; Wrote the paper.

Robert Middleton: Conceived and designed the experiments; Performed the experiments; Analyzed and interpreted the data.

### Funding statement

This work was funded by Firstkind Limited as part of a grant from the Technology Strategy Board (now Innovate UK), from the Biomedical Catalyst Late Stage funding scheme. The funding source did not play a role in the data collection, analysis, interpretation or writing of the manuscript.

### Competing interest statement

The authors declare the following conflict of interests: Thomas Wainwright and Robert Middleton are shareholders in Healthdecoded Ltd. Healthdecoded Ltd has performed consultancy activities for Firstkind Ltd who manufacturer the geko™ device. There is potential in the future, for Healthdecoded Ltd to be provided with an option to buy shares in Sky Medical Technology (the parent company of Firstkind Ltd). Louise Burgess declares no conflict of interest.

### Additional information

The clinical trial described in this paper was registered at ClinicalTrials.gov under the registration number NCT01935414.

## References

[bib1] Broderick B.J., Kennedy C., Breen P.P., Kearns S.R., ÓLaighin G. (2011). Patient tolerance of neuromuscular electrical stimulation (NMES) in the presence of orthopaedic implants. Med. Eng. Phys..

[bib2] Broderick B.J., O'Briain D.E., Breen P.P., Kearns S.R., Olaighin G. (2010). A pilot evaluation of a neuromuscular electrical stimulation (NMES) based methodology for the prevention of venous stasis during bed rest. Med. Eng. Phys..

[bib3] Browse N.L., Negus D. (1970). Prevention of postoperative leg vein thrombosis by electrical muscle stimulation. An evaluation with 125I-labelled fibrinogen. Br. Med. J..

[bib4] (2017). CONSORT Transparent Reporting of Trials.

[bib5] Dejode L.R., Khurshid M., Walther W.W. (1973). The influence of electrical stimulation of the leg during surgical operations on the subsequent development of deep-vein thrombosis. Br. J. Surg..

[bib6] Den Hartog Y.M., Mathijssen N.M.C., Vehmeijer S.B.W. (2013). Reduced length of hospital stay after the introduction of a rapid recovery protocol for primary THA procedures. Acta Orthop..

[bib7] Den Hartog Y.M., Mathijssen N.M.C., Vehmeijer S.B.W. (2015). Total hip arthroplasty in an outpatient setting in 27 selected patients. Acta Orthop..

[bib8] Doran F.S.A., White H.M. (1967). A demonstration that the risk of postoperative deep venous thrombosis is reduced by stimulating the calf muscles electrically during the operation. Br. J. Surg..

[bib9] Faghri P.D., Van Meerdervort H.F., Glaser R.M., Figoni S.F. (1997). Electrical stimulation-induced contraction to reduce blood stasis during arthroplasty. IEEE Trans. Rehabil. Eng..

[bib10] Griffin M., Bond D., Nicolaides A.N. (2016). Measurement of blood flow in the deep veins of the lower limb using the geko^TM^ neuromuscular electro-stimulation device. Int. Angiol..

[bib11] Gromov K., Kjaersgaard-Andersen P., Revald P., Kehlet H., Husted H. (2017). Feasibility of outpatient total hip and knee arthroplasty in unselected patients. Acta Orthop..

[bib12] Husted H., Holm G., Jacobsen S. (2008). Predictors of length of stay and patient satisfaction after hip and knee replacement surgery: fast-track experience in 712 patients. Acta Orthop..

[bib13] Husted H., Lunn T.H., Troelsen A., Gaarn-Larsen L., Kristensen B.B., Kehlet H. (2011). Why still in hospital after fast-track hip and knee arthroplasty?. Acta Orthop..

[bib14] Ibrahim M.S., Twaijj H., Giebaly D.E., Nizam I., Haddad F.S. (2013). Enhanced recovery in total hip replacement. Bone Jt. J..

[bib15] Jorgensen C.C., Kehlet H. (2017). Thromboembolic and major bleeding events in relation to perioperative bridging of vitamin K antagonists in 649 fast-track total hip and knee arthroplasties. Acta Orthop..

[bib16] Kerchner K., Fleischer A., Yosipovitch G. (2008). Lower extremity lymphedema: update: pathophysiology, diagnosis, and treatment guidelines. J. Am. Acad. Dermatol..

[bib17] Summers J.A., Clinch J., Radhakrishnan M., Healy A., McMillan V., Morris E., Rua T., Ofuya M., Wang Y., Dimmock P.W., Lewis C., Peacock J.L., Keevil S.F. (2015). The geko^TM^ electro-stimulation device for venous thromboembolism prophylaxis: a NICE medical technology guidance. Appl. Health Econ. Health Pol..

[bib18] Tucker A., Maass A., Bain D., Chen L.-H., Azzam M., Dawson H., Johnston A. (2010). Augmentation of venous, arterial and microvascular blood supply in the leg by isometric neuromuscular stimulation via the peroneal nerve. Int. J. Angiol..

